# Drivers of household demand for cooking energy: A case of Central Uganda

**DOI:** 10.1016/j.heliyon.2022.e09118

**Published:** 2022-03-15

**Authors:** Edmond Nyuyki Mainimo, Daniel Micheal Okello, Wilson Mambo, Basil Mugonola

**Affiliations:** a Department of Rural Development and Agribusiness, Faculty of Agriculture and Environment, Gulu University, P.O Box 166, Gulu, Uganda; b Technology Development Centre, Uganda Industrial Research Institute, P.O Box 7086 Kampala, Uganda

**Keywords:** Cooking energy demand, Charcoal, Briquettes, Firewood, Uganda

## Abstract

In Uganda, the total primary household energy consumption is mainly biomass. Lack of empirical information remains a daunting challenge to operationalization of strategies and policies aimed at reducing unsustainable energy use. This study specifically determined household demand for different cooking energy sources (briquettes, charcoal and firewood). Data were collected from a sample of 235 households with the help of pretested structured questionnaires. Data were analyzed using descriptive statistics and Seemingly Unrelated Regression (SUR). From the econometric analysis, results revealed that briquettes demand was significantly influenced by fuel expenditure, briquette price, gender of household head, main occupation and source of fuel. Further, charcoal demand was influenced by fuel expenditure, household size, charcoal price, district and fuel restriction. On the other hand, demand for firewood was influenced by household income, firewood price, fuel expenditure, household size and source of the fuel. From our findings, it is recommended that tailored trainings on sustainable exploitation of biomass resources for energy supply should be conducted across the country. In addition, there is need for such trainings to target women, who are the decision makers with respect to household energy supply. Lastly the study recommends the need for low-cost briquetting technologies that would reduce its price to levels that are competitive to charcoal and firewood.

## 1 Introduction

Global access to energy is necessary to enhance human survival and to achieve economic, social as well as environmental aspects of human development [1]. In recent times, there has been a rapid increase in the number of countries exploiting biomass opportunities for energy provision; a reason why it has presented itself as a more attractive and promising option compared to other renewable energy sources [2, 3, 4]. Globally, the number of people depending on traditional biomass as a source of heating and cooking fuel was estimated at 2.4 billion people in 2002 and is expected to reach 2.6 billion in 2030 (an 8% increase). In sub-Saharan Africa, over half of all urban households depend on either firewood, charcoal or wood waste to meet their cooking needs [5].

In East Africa, the demand for energy is also on the rise, for households, businesses and industry. Increasing populations, intense deforestation, and expanding economies have led to increasing fuel prices and shortages, which often hits hardest the households and businesses most in need. Fuel prices have increased steadily throughout East Africa in recent times. The past few years alone have seen significant hikes in the prices of cooking fuels including Liquefied Petroleum Gas (LPG), electricity and kerosene [6, 7, 8].

In most developing countries including Uganda, there has been a rapid growth in demand for energy with current demand superseding supply in most cases and energy scarcity hindering sustainable economic transformation. In 2010, the government of Uganda asserted that energy poverty was a key impediment to economic and social transformation. Mukwaya [9] agreed to this by stating that energy insecurity is a key obstacle for Uganda's economic advancement. According to Ferguson [10], biomass accounts for 90% of the total energy used in Uganda. In terms of cooking energy, Global Alliance for Clean Cook stoves (GACC) as cited by Price [11], reported that cooking fuels in Uganda comprise of unprocessed biomass used by over 85% of the population. Charcoal is used by 13% of the population dwelling in majorly urban and peri urban settings with over 10% of the total charcoal sold in urban centers being discharged as wasted charcoal dust [12]. LPG and kerosene are used by a very small percentage of households (below 0.5% each) while about 0.8% uses a mix of cooking fuels from microenterprises [13].

Household cooking energy is the energy utilized for cooking and does not include energy used for food production, food processing and preparation before purchase. It is a major part of the total energy consumed at home [7]. According to Mahoro *et al.* [14], the household sector tends to experience the most pronounced changes in its pattern of fuel use overtime. This shift is typically from biomass to kerosene, gas and finally electricity in case of need for specialized cooking [15]. The quantity and type of energy used in cooking depends on income, availability of fuel, cooking behavior and efficiency of the appliances [16, 17]. According to Tucho & Nonhebel [18], the composition of fuel types determines the amount of biomass energy used by households. This composition of fuel increases with relatively less biomass energy when household energy demand increases as stipulated by the energy ladder model [19, 20].

Despite current policy prioritization, lack of empirical information remains a daunting challenge to operationalization of policies and strategies regarding sustainable use of cooking energy sources. One of the critical constraints in this regard is the lack of clear and empirical information on the drivers of demand for cooking energy sources. This information is indispensable in the identification of solutions and guiding priorities of action. In this study, we contribute to cooking energy literature by analyzing the drivers of household demand for cooking energy in Uganda.

The rest of the paper is structured as follows: the next section (section 2) presents a review of literature on the theories and methodologies for understanding household cooking energy demand, this is followed by the materials and methods (section 3), presentation of results (section 4), and discussions of results (section 5). Finally, the conclusions including limitations to the study, policy recommendations and suggestions for further research are presented the last section.

## 2 Literature review

Various research efforts have been focused on household cooking energy sources; a subject, which is closely related to cooking energy demand. Two main theories have been advanced in an attempt to explain household energy choices. The ‘‘energy ladder model’’ theory postulates that households gradually climb an energy ladder starting with traditional energy sources, and progressing to commercial fuels and finally to the use of advanced fuels like electricity [7, 21]. The shift through these three phases is steered by prices of fuels and household incomes. The model assumes a linear progression pattern of households as they move along the imaginary energy ladder, switching completely from traditional fuels as their incomes increase. However, recent studies have revealed that as affluence increases, traditional fuels are not totally discarded but are rather used in a mix with other energy sources and that income does not influence household fuel use [22, 23] thus pointed out these as limitations with the energy ladder model. The limitations of the “energy ladder” model led to the proposition of the fuel stacking model [24]. The “fuel stacking” model states that the switch by households to using clean energy is not linear, but rather households just increase the number of energy fuels used without necessarily stopping the use of old ones completely [7]. Here, energy use patterns of households are guided by several factors and not only income [25].

A broad array of scientific literature exists on energy use patterns in developing countries, but most of these previous studies are based on descriptive statistics. However, a few of them applied econometric analytical approaches in trying to understand the drivers of household energy cooking choice, cooking energy demand or both. Econometric studies on this subject can be classified into three groups based on the focus of the study. The first category focused on different energy sources [26, 27, 28]. The second category focused on household energy demand [29, 30] while the third category considered both choice and demand for household energy demand [31, 32, 33]. Empirically, this study took into consideration previous studies including [7, 29, 32, 34, 35, 36] that focused on household use of cooking fuels.

These studies used several analytical approaches with most of them applying Chi-square analysis, multiple regression, multivariate Probit regression, multinomial logit and ordered Probit models. Studies on the factors that determine the demand for cooking energy often applied the ordinary least squares (OLS) technique since the response variable (fuel quantity) is assumed to be continuous [34, 36]. The findings of these studies indicate that household fuel use is influenced by factors including income, prices, gender of household head, household size, expenditure, location, education, type of dwelling, and distance to the fuel source. Suffice to note that the dimension and extent of influence varied across the different cooking energy types. To the best of our knowledge, there is a paucity of empirical studies on energy demand and the only published works on Uganda in the area of cooking fuel choice or demand are those of [36, 37, 38, 39]. Tabuti *et al.* [38] conducted a study to determine preferred firewood species, their harvesting and consumption patterns in Bulamogi-Uganda. Their study revealed that firewood was abundant but declining in quantity. They attributed this to increasing demands generated by the growing population of Bulamogi, and the growing need for charcoal. In another study, Lee [36] sought to test the relationship between household socioeconomic characteristics and the use of various cooking fuels, as well as the factors that increase the chances of switching from solid fuels to transitional fuels in Uganda. The study showed that electricity consumption has a direct relationship with income. Provision of public infrastructure, income and education were key variables that could be used to reduce household use of solid fuels. Additionally, Drazu *et al.* [37] investigated the state and breakdown of energy consumption in Uganda. Their findings suggested a strong demand for energy, which is highly dominated by solid fuel sources. Most households used firewood and charcoal for cooking while electrical energy was majorly used for lighting and entertainment. Bamwesigye & Darkwah [39] in their investigation explored the forest wood biomass in Uganda and examined the functions, values and trends. The findings indicated that the value of wood charcoal production increased from 10% to 13%. They noted that everything being equal, energy was pivotal to sustainable development and poverty eradication in Uganda. It is therefore clear that previous studies had different areas of focus from this current study.

Based on this review and field data, several factors were used to ascertain their influence on the demand for charcoal dust briquettes, charcoal and firewood in Central Uganda. Considering that the socioeconomic factors affecting household demand for cooking fuels vary from region to region, it becomes apparent that those mentioned in other studies cannot be similarly applicable to Central Uganda. This study therefore sought to ascertain the drivers of demand for biomass-based cooking energy types (briquettes, charcoal and firewood) in Central Uganda.

## 3 Materials and methods

### 3.1 Study area

**Figure 1:**
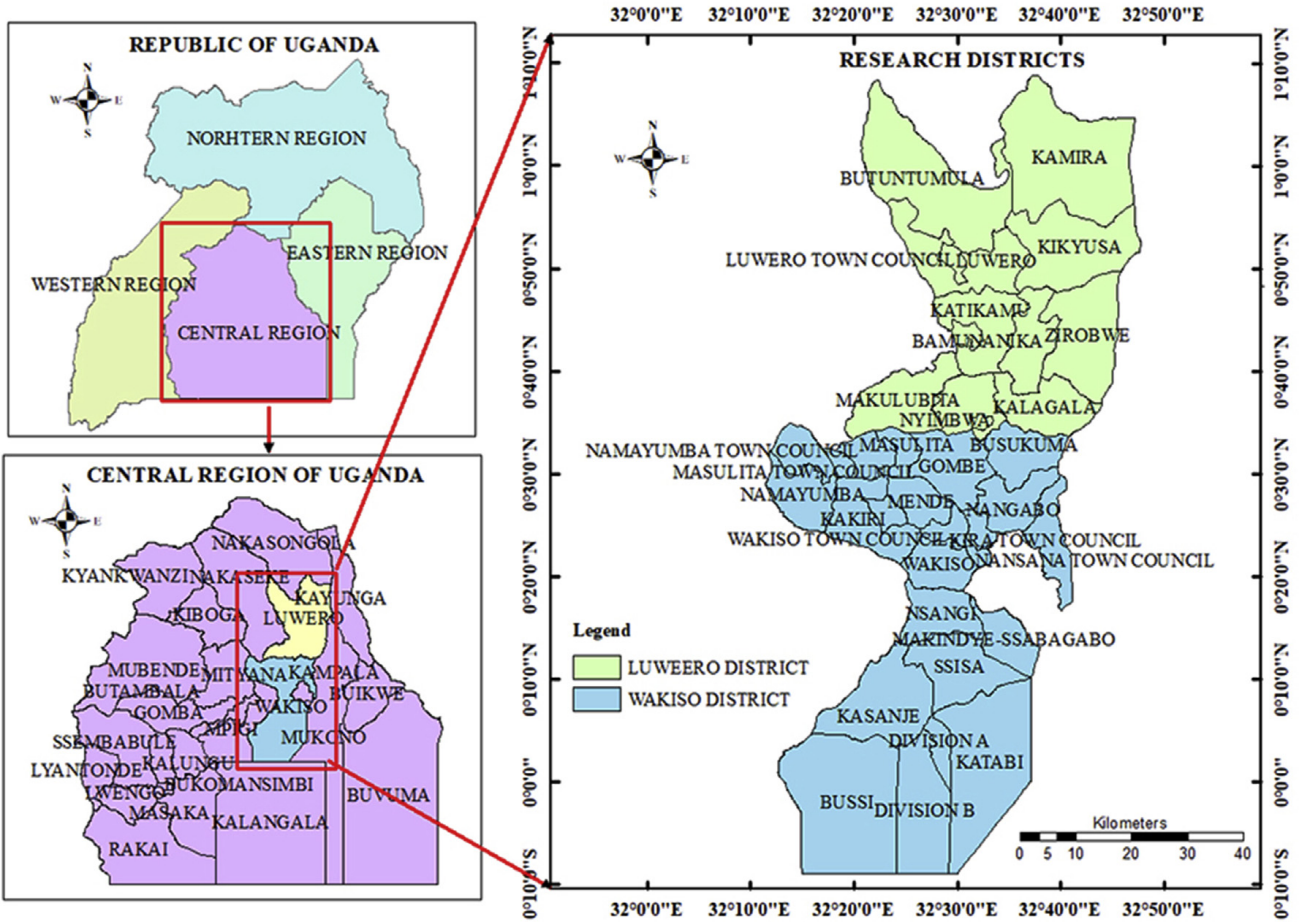
Map of the study area: Luwero and Wakiso and their relative location in Uganda. (The map was developed by the researchers using Arc-GIS software).

This study was conducted in Luwero and Wakiso districts of Central Uganda (Figure 1). These two districts were conveniently chosen because of their predominance in the use briquettes, charcoal and firewood for cooking. Luwero district lies North of Kampala the capital and largest city in Uganda, between latitude 2^0^ north of the Equator and east between 32^0^ to 33^0^. Luwero is bordered by Nakasongola district to the North, Kayunga district to the east, Mukono district to the South East, Wakiso district to the south, and Nakaseke district to the west. Luwero has a total area of approximately 2,577.49 square kilometers and is divided into ten sub counties of Bamunanika, Kalagala, Kamira, Kikyusa, Zirobwe, Makulubita, Nyimbwa, Butuntumula, Katikamu and Luwero. It has mean temperatures ranging between 8 °C and 35 °C. The rainfall is well distributed throughout the year, with the average annual rainfall being 1,300mm. The populace is mainly engaged in agricultural and livestock production. Major horticultural crops grown include tomatoes, pineapples and cabbage [40].

Wakiso district surrounds Kampala and boarders Mukono in the east, Mubende and Mpigi in the west, Luwero in the north and Kalangala in the south. The district is the second most populated district in Uganda and covers a total area of 2,807.75 square kilometers. The Climate in Wakiso is warm and wet with relatively high humidity. The rainfall is bi-modal with mean annual rainfall of 1,320mm though in many areas of the lake zone is between 1,750mmn – 2000mm. The minimum surface air temperature of the district is 11.0 °C while the maximum is 33.3 °C. The economic activities in Wakiso district include fishing on Lake Victoria, poultry feeds, and agriculture with emphasis on food crops like sweet potatoes, beans, Irish potatoes and soya beans. Cash crops include coffee and cotton while fruits and vegetables widely grown are tomatoes, onions and cabbage [40].

### 3.2 Sampling design

A cross sectional survey design was used to collect primary data from the households. The target study population was households that use biomass briquettes a cooking energy source in Luwero and Wakiso districts of Central Uganda. Multi-stage sampling procedure was used in the selection of a representative sample. In the first stage, two districts (Luwero and Wakiso) were purposively sampled based on their extent of use of these cooking fuels and proximity to the Kampala city. In the second stage, five sub counties/town councils (Luwero Town council, Katikamu, Wobulenzi, Gombe and Nangabo) were selected purposively because of the relative concentration of users. In the final stage, simple random sampling was used to select at least 46 households from each sub-county to give a total of 235 households that were subsequently interviewed.

### 3.3 Data collection

Data were collected through the administration of pretested structured questionnaires in face-to-face interviews. Prior to data collection, informed consent was sought from all participants and only those that consented were interviewed. The informed consent was prepared following guidelines provided by Gulu University Research and Ethics Committee (GUREC); the committee that approved the study (Reference No: GUREC-037-20). The first part of the questionnaire captured data on household demographic and socio-economic characteristics while the second part focused on household cooking fuel including monthly quantity of fuel used, and monthly fuel expenditure.

### 3.4 Data analysis: the SUR model

The individual demand equation of each cooking energy source is specified as a function of key drivers, including fuel expenditure, price, household size, district, gender, source of fuel, fuel restriction, household income, main occupation and education level (Table 1). The individual demand function for charcoal, briquettes and firewood is as specified in Eq. (1)

(1) Y_i_ = β_0_ + β _i_ X_i_ + μ_i_

Y_i_ = Quantity of Cooking fuel demanded, β_0_ = Regression coefficient, β_i_ = a vector of parameters associated with each of the explanatory variables, X _i_ = a vector of explanatory variables, μ _i_ = Error term or random disturbance [36].

**Table 1:** Variables used in the seemingly unrelated regression model of drivers of household's demand for briquettes, charcoal and firewood.

Variable	Description/Measurement of variable	Expected sign
Gen	Sex of the household head (Male = 1, Otherwise)	+/-
HsInc	Household monthly income status (Uganda Shillings)	+/-
Hsize	Number of persons in a household (Numbers)	+
Educ	Years spent in school by household head (Years)	+/-
FuelExpd	Average monthly fuel expenditure (Uganda Shillings)	+
Price	Unit price per Kg of charcoal dust briquettes (Uganda Shillings)	-
Occup	Occupational status (1 = Farmer, 2 = Businessman,	+/-
	3 = Casual Worker 4 = Salaried (Employed)	
District	District of household (1 = Luwero, 0 = Wakiso)	+/-
F.Source	Source of the fuel (1 = Own production, 0 = Market place)	+/-
Restric	Household restriction of fuel use (1 = Yes, 0 = No)	-

**Note:** The expected signs in Table 1 indicate a positive, negative or mixed effect on household fuel demand.

The demand equations for this cooking fuels are estimated efficiently using the SUR model. Given that cooking energy sources are considered as substitutes or complements to one another, there is a strong possibility that a strong interrelationship exists between the cooking energy demand function for charcoal, briquettes and firewood, primarily due to correlation between their disturbances. Taking this into account, estimating each equation using the OLS will give us consistent but inefficient coefficients. The seemingly unrelated model in this case is appropriate as the estimated coefficients are both consistent and effective. Therefore, the SUR model of the demands for the cooking energy sources can be specified as a set of three demand equations (briquettes, charcoal, firewood) that has contemporaneous cross-equation error correlation (the error terms in the equations are correlated). The SUR demand equations used in the study are presented in Eqs. (2), (3), and (4).

(2) Y_i_ _ _Briquettes_ = β_0_ + β_1_Fuelxpd+ β_2_Hsize+ β_3_ P_ _Briquettes_+ β_4_District+ β_5_Gen + β_6_F.Source__Briquettes_ + Β_7_Occup+ β_8_Restric + β_9_Educ + β_10_HsInc + μ_i_

(3) Y_i_ _ _Charcoal_ = β_0_ + β_1_Fuelxpd+ β_2_Hsize+ β_3_ P_ _Charcoal_+ β_4_District+ β_5_Gen + β_6_F.Source__Charcoal_ + Β_7_Occup+ β_8_Restric + β_9_Educ + β_10_HsInc + μ_i_

(4) Y_i_ _ _Firewood_ = β_0_ + β_1_Fuelxpd+ β_2_Hsize+ β_3_ P_ _Firewood_+ β_4_District+ β_5_Gen + β_6_F.Source__Firewood_ + Β_7_Occup+ β_8_Restric + β_9_Educ + β_10_HsInc + μ_i_

## 4 Results

### 4.1 Demographic and socio-economic characteristics of households

The results in Table 2 showed that majority (69%) of the households were male-headed. In terms of marital status, most (65%) of the briquette users were married. The chi-square results for marital status were statistically significant at 5% indicating that there was significant association in marital status in the two districts.

**Table 2:** Descriptive statistics (Pearson Chi-square analysis) for demographic and socio-economic characteristics of briquette users in Luwero and Wakiso districts.

Variable	Frequency (Percentage)			χ^2^	Sig.
	Luwero N = 137	Wakiso N = 98	Overall		
**Gender**					
Male	90 (66)	73 (74)	163 (69)	2.08	0.149
Female	47 (34)	25 (26)	72 (31)		
**Marital Status**					
Married	83 (61)	69 (70)	152 (65)	9.49∗∗	0.023
Unmarried	54 (39)	29 (30)	83 (35)		
**Main occupation**					
Farmer	48 (35)	6 (6)	54 (23)	28.99∗∗∗	0.000
Business	44 (32)	36 (37)	80 (34)		
Casual worker	13 (10)	16 (16)	29 (12)		
Employee	32 (23)	40 (41)	72 (31)		
**Cooking place**					
Indoor	98 (72)	69 (70)	167 (71)	0.035	0.851
Outdoor	39 (28)	29 (30)	68 (29)		
**Income Status**					
1=<250,000	57 (42)	27 (28)	84 (36)	10.60∗∗	0.014
2 = 250000-499000	21 (15)	25 (26)	46 (19)		
3 = 500000-1000000	56 (41)	38 (38)	94 (40)		
4=>1000000	3 (2)	8 (8)	11 (5)		
**Residential location**					
Rural	59 (43)	0 (0)	59 (25)	56.35∗∗∗	0.000
Urban	78 (57)	98 (100)	176 (75)		

Source: Field survey, (2019); Number of respondents = 235, χ^2^ denotes Pearson's chi-square coefficient; ∗∗∗ & ∗∗ implies p < 0.01 and p < 0.05.

In terms of occupation, most (34%) of the household heads reported business as the main occupation. In Luwero district, 35% of the households were farmers, 32% were businesspersons, 10% were casual workers and 23% were paid employees. On the other hand, 6% of the users in Wakiso district were farmers, 37% businesspersons, 16% casual workers and 41% were paid employees. The chi-square results revealed significant association (p < 0.01) between main occupation and household district location, indicating that households whose main occupation was business were more in Wakiso district than in Luwero district. With regards to cooking place, more households (71%) cooked indoors. In addition, a greater proportion of the households reported a monthly income of at least UGX 500,000. However, a few (5%) had over a million Uganda shilling per month. The chi square test for association between household income and district was statistically significant at 5% implying that there was a significant difference in the distribution of household income in Luwero and Wakiso district.

In terms of residential location, majority (75%) of the households were located in urban areas. In Luwero district, 43% of the households were in rural areas while 57% were in the urban areas. Meanwhile in Wakiso, all the households were situated in the urban areas. The chi-square results showed that residential location was statistically significant. This therefore indicated that there were more urban households in Wakiso district than Luwero district.

The result of the chi-square test for gender and cooking place revealed that they were statistically insignificant; indicating that the distribution of these characteristics in the two districts were almost similar. The results in Table 3 indicated that the mean age of the household head for Luwero district was greater (46 years) than for Wakiso district (39 years). The results of the t-test analysis for age was statistically significant at 1% indicating that household heads in Luwero district were older than those in Wakiso district. In terms of household size, the mean was approximately the same (5members) for the two districts.

**Table 3:** t-test for equality of means in age, household size and education level of briquette users by district.

Variable	Mean		Mean difference	Sig.
	Luwero (N=(137))	Wakiso (N=(98))		
Age	46.109	39.398	6.711∗∗∗	0.000
Household size	5.459	5.081	0.378	0.299
Education level	9.788	11.693	-1.905∗∗∗	0.000

Source: Field survey, (2019); Number of respondents = 235; ∗∗∗ Significant at 1% level.

Furthermore, the mean education level of the household head was greater for Wakiso than Luwero district. Following the t-test analysis, education level was statistically significant at 1%. This implied that household heads in Wakiso district were more educated compared to those in Luwero district.

### 4.2 Drivers of household cooking energy demand

The Breusch-Pagan test of independence confirmed existence of correlated error terms of the three demand equations. The adjusted – R^2^ (0.685) showed that 68% of the variation in the demand for briquettes was explained by the independent variables. The Variance Inflation Factor (VIF) test had mean VIF of 1.3. All the variables in the briquette demand model had VIF values of less than 3 which is highly acceptable and thus indicating absence of multicollinearity between the explanatory variables. Empirical results from the SUR model revealed that fuel expenditure (p = 0.000), gender (female) (p = 0.012), source of fuel (own production) (p = 0.002), briquette price (p = 0.000) and business as main occupation (p = 0.005) were statistically significant and hence exert influence on the demand for briquettes as a cooking fuel (Table 4). Fuel expenditure, gender and source of fuel had positive influence on the demand for briquettes. On the other hand, briquette price and main occupation had negative influence.

**Table 4:** SUR model results for drivers of demand for cooking energy sources in Luwero and Wakiso districts.

Variables	Briquettes		Charcoal		Firewood	
	Coefficient	p ≥ t	Coefficient	p ≥ t	Coefficient	p ≥ t
Constant	-1.987	0.030	0.765	0.002	-0.311	0.052
Expenditure	0.802	0.000∗∗∗	0.432	0.000∗∗∗	0.288	0.000∗∗∗
Household size	0.010	0.590	0.012	0.043∗∗	0.221	0.000∗∗∗
Price	-0.464	0.000∗∗∗	-0.678	0.000∗∗∗	-0.001	0.000∗∗∗
Gender	0.215	0.012∗∗	-0.003	0.923	0.041	0.868
District	-0.045	0.602	-0.097	0.000∗∗∗	0.120	0.122
Source of fuel	0.283	0.002∗∗∗	0.010	0.789	0.185	0.087∗
Main Occupation	-0.113	0.005∗∗∗	-0.005	0.646	-0.014	0.875
Fuel restriction	-0.001	0.557	-0.112	0.024∗∗	-0.017	0.668
Education level	0.004	0.965	0.077	0.090∗	0.041	0.668
Income Status	-0.267	0.177	0.026	0.402	0.155	0.078∗
No. of Observations	235		235		235	
Prob > F	0.000		0.000		0.000	
Adjusted R^2^	0.685		0.786		0.870	
Mean VIF	1.30		1.25		1.55	

**Source:** Survey data, 2019 ∗∗∗; ∗∗ and ∗ denote significance at 1%; 5% and 10% respectively.

The adjusted – R^2^ (0.786) showed that 78 % of the variation in the demand for charcoal was explained by the independent variables. The VIF test had mean VIF of 1.25. All the variables in the charcoal demand model had VIF values of less than 4, indicating the that there is no multicollinearity between the explanatory variables. The results indicated that the demand for charcoal was influenced by fuel expenditure (p = 0.000), charcoal price (p = 0.000), fuel restriction (p = 0.024), household size (p = 0.043), district (Luwero) (0.000) and education level (p = 0.090). Household size, fuel expenditure and education level of household head positively influenced the demand. Meanwhile, fuel restriction, district and price of charcoal had a negative impact.

For firewood demand, the adjusted – R^2^ (0.870) showed that 87 % of the variation in the demand for firewood was explained by the independent variables. The VIF test had mean VIF of 1.55. All the variables in the firewood demand model had VIF values of less than 4 which is highly acceptable as evidence of no multicollinearity between explanatory variables. Empirical results showed that fuel expenditure (p = 0.000), firewood price (p = 0.000) household size (p = 0.006), source of fuel (p = 0.087) and household income status (p = 0.078) had influence on firewood demand (Table 4). Fuel expenditure, source of the fuel, household income level and household size influenced firewood demand positively while price had a negative effect.

## 5 Discussions

This study revealed that as the fuel expenditure of the household increased, the demand for briquettes, charcoal and firewood increased. A one Uganda shilling rise in fuel expenditure, increased the proportion of briquette quantity demanded by 0.802 and vice versa. For charcoal demand, a unit increase in expenditure increased the quantity of charcoal demanded by 0.432. Additionally, a one Uganda shilling increase in fuel expenditure increased the quantity of firewood demanded by 0.288. The effect of fuel expenditure of demand could be explained in relation to the number of meals prepared in households and also in relation to the relative availability of the different energy sources. The higher the number of meals cooked, the higher the fuel expenditure and therefore the higher the chances of demanding for more of these cooking fuels.

It was equally observed that an increase in household income culminated in a corresponding increase in the demand for firewood *ceteris paribus*. A one Uganda shilling increase in income increased firewood demand by 0.155. Household income was not a significant factor in the case of demand for briquettes and charcoal. The income elasticities for firewood were positive, indicating these cooking fuels are not inferior goods in the study area. The increase in demand could be explained by high dependence on charcoal and firewood in the study area because of easier access and affordability compared to other forms of energy including electricity and Liquefied petroleum gas. Furthermore, this increase could be because in Luwero and Wakiso, there are food dishes like *matooke* (East African highlands bananas) that for some cultural reasons are preferably cooked using firewood. Remigios [41] argued that most villages in rural Zimbabwe prefer dishes like sadza (thick maize meal porridge) to be cooked using firewood. This argument is in line with the findings of Ifegbesan *et al.* [34] in Kano state Nigeria who argued that firewood gives flavor to food cooked with it. Additionally, Makonese & Ifegbesan [42] noted that households do not automatically switch their demand from traditional fuels to more modern and sophisticated energy sources with an increase in disposable income. This finding was however contrary to the energy ladder hypothesis of advancement in energy source with a rise in income [21]. Similarly, Bisu *et al.* [7] reported that the use of charcoal and firewood gradually decreased with increase in income in Bauchi Metropolis, Nigeria.

Furthermore, a unit increase in household size resulted in a 0.012 and 0.221 increase in the quantity demanded of charcoal and firewood respectively. The increase in family size due to visitations and the attendant increase in the number of children increased the energy demands, food, shelter, clothing, health, education and other needs. This makes cooking with advanced fuels like LPG uneconomical and may necessitate consumption of relatively cheaper fuels (charcoal and firewood). On the other hand, increase in household size equally indicated an increase in the labor supply for firewood collection especially in the rural areas [29]. The finding is comparable to the results of Obrumah *et al.* [43] who found out that large household size is a barrier for households to switch to clean and modern energy sources in Ghana. This result is also in line with a previous Nigerian study by [44] which noted that larger households used more quantities of firewood in the urban areas of Enugu State.

In addition, a unit increase in the price of briquettes, charcoal and firewood decreased the quantity demanded for the cooking fuels by 0.464, 0.678 and 0.001 respectively. The estimated price elasticity in absolute value was below unity, implying that demand for briquettes was inelastic. A reduction in the availability of briquettes probably due to scarcity especially in the rainy seasons for example may cause a price hike of the fuel, a reduction in the frequency of use of briquettes for cooking and thus the quantity demanded. This finding generally conforms to economic theory that depicts an inverse relationship between quantity demanded and price. This finding is in line with the findings of Sagbo [45] who reported that briquette users in Haiti were very sensitive to the price and tended to demand lesser as the price increased.

It is worth noting that households in Luwero district demanded less of charcoal compared to their counterparts in Wakiso district. This is probably because of the differences in the level of urbanization in the two districts. Households in Luwero district are more likely to freely access firewood to complement their cooking energy mix thereby reducing on their relative demand for charcoal [46].

Households headed by females tended to demand more briquettes than those headed by their male counterparts. This result is obvious in the context of a developing country like Uganda where women are the key decision makers in cooking and other household chores. Moreover, in the study area there are women associations that are actively involved in briquette production. Consequently, it is mostly women who are aware of the benefits and disbenefits associated with the different cooking energy sources, and would usually opt for cleaner options. This finding is in agreement with Bahadur *et al.* [32] who showed that female headed households were more likely to opt for modern cooking energy fuels in cases where their use is relatively economical.

Furthermore, there was the likelihood of households that produces their own briquettes and firewood to consume more than their counterparts who procure from the market. This could be attributed to the fact that buying from the market entails both cost of the product and transportation cost. Briquettes made from home are relatively cheaper since such households are able to utilize relatively available biomass for producing the required briquettes. This finding is comparable to that of Imran *et al*. [47] who asserted that the choice of fuel use is significantly affected by the distance to the source of the fuel.

Demand for briquette by households headed by businesspersons is likely to be lower than that of those headed by their counterparts (casual worker, peasant farmer, and employee) in the study area. This behavior is probably because of an improvement in income, which tend to place them in a higher social class causing them to demand more of better fuels like LPG. This is comparable to the findings of Imran *et al*. [47], who stated that people tend to demand more of improved cooking energy sources as their income increased. This could also be explained by the fact that businesspersons tend to have less time for other activities therefore demand for faster fuels such as LPG.

With restriction to the type of fuel used in the household, the demand for charcoal tends to decrease. The result could be attributed to the fact that for main household dwelling units that are rented, the tenant must abide by the landlord occupancy rules. In the case where the dwelling unit is a modern type house, the landlord might be concerned about the smoke produced by the charcoal that can stain the wall and roofs. The findings of the study are comparable to the findings of [7] who reported that dwelling ownership status (fuel restriction) significantly influenced the consumption of kerosene for households in Bauchi Metropolis, Nigeria. Based on these findings, it can be inferred that the influence of the fitted explanatory variables differed across the demand for specific cooking energy fuels in Luwero and Wakiso districts of Uganda.

## 6 Conclusions and policy recommendations

The study sought to fill a knowledge void on the drivers of household demand for cooking energy sources. The principal drivers of demand for briquettes are fuel expenditure, gender and source of fuel (homemade versus purchased) and main occupation. For charcoal demand, they included fuel expenditure, household size, price, dummy of district and restriction to fuel use. In case of our study, firewood demand was influenced by fuel expenditure, household size, fuel price, source of the fuel and income status. The operationalization of policies and strategies that seek to reduce the use of unsustainable cooking energy sources and to promote the use of alternative options can be based on these findings. For instance, in order to increase the use of clean energy sources including briquettes, there should be deliberate efforts to target direct users such as women who are at the forefront in decision making when it comes to household energy needs. There is need to focus on briquetting technologies that are able to reduce cost and consequently reduce the price to levels that make it competitive with charcoal and firewood.

It is worth noting that this study only focused on household demand for cooking energy sources. Institutional consumers including restaurants and schools were not considered. Further, the study focused on demand for biomass-based cooking energy sources. The drivers of demand for alternative cooking energy sources like biogas, solar and electricity was not ascertained. The findings of this study are therefore not applicable to situations of institutional demand for cooking energy, neither can it be applied to situations of other cooking energy sources which were not the focus of the study. We recommend further research to focus on institutional drivers of cooking energy demand and/or drivers of cooking energy sources not included in this study.

## Declarations

### Author contribution statement

Edmond Nyuyki Mainimo: Conceived and designed the experiments; Performed the experiments; Analyzed and interpreted the data; Wrote the paper.

Daniel Micheal Okello, Basil Mugonola: Conceived and designed the experiments; Analyzed and interpreted the data; Wrote the paper.

Wilson Mambo: Conceived and designed the experiments; Performed the experiments; Wrote the paper.

### Funding statement

This work was funded by the MasterCard Foundation grant to the Regional Universities Forum for Capacity Building in Agriculture (RUFORUM) and Gulu University, under the project “Transforming African Agricultural Universities to meaningfully contribute to Africa's growth and development (TAGDev)”.

### Data availability statement

Data will be made available on request.

### Declaration of interests statement

The authors declare no conflict of interest.

### Additional information

No additional information is available for this paper.
